# Biophysical Equations and Pressure Probe Experiments to Determine Altered Growth Processes after Changes in Environment, Development, and Mutations

**DOI:** 10.3390/plants11030302

**Published:** 2022-01-24

**Authors:** Joseph K. E. Ortega

**Affiliations:** Department of Mechanical Engineering, University of Colorado Denver, Denver, CO 80217-3364, USA; joseph.ortega@ucdenver.edu

**Keywords:** plant growth response, fungal growth response, augmented growth equations, pressure probe, dimensionless numbers, Π parameters

## Abstract

Expansive growth is a culmination of many biological processes. It is fundamental to volume growth, development, morphogenesis, sensory responses, and environmental responses of plants, fungi, and algae. Expansive growth of walled cells and plant tissue can be accurately described by a set of three global biophysical equations that model the biophysical processes of water uptake, wall deformation, and turgor pressure. Importantly, these biophysical equations have been validated with the results of pressure probe experiments. Here, a systematic method (scheme) is presented that iterates between analyses with the biophysical equations and experiments conducted with the pressure probe. This iterative scheme is used to determine altered growth processes for four cases; two after changes in the environment, one after a change in development, and another after changes by mutation. It is shown that this iterative scheme can identify which biophysical processes are changed, the magnitude of the changes, and their contribution to the change in expansive growth rate. Dimensionless numbers are employed to determine the magnitude of the changes in the biophysical processes. The biological meaning and implication of the biophysical variables in the biophysical equations are discussed. Further, additional sets of global biophysical equations are presented and discussed.

## 1. Introduction

Volumetric growth rates of plants and fungi depend on the expansive growth rates of individual plant and fungal cells (cells with walls). The expansive growth rate of the individual walled cell is a function of the rates of two interrelated and simultaneous biophysical processes: *net* water uptake and wall deformation. The rates of these biophysical processes are altered by changes in environment, development, and mutations. The determination of whether the net water uptake rate and/or the wall deformation rate are altered, and how they affect expansive growth rate, is important to a better understanding of how plant and fungal cells respond to these changes. It may be that some of the alterations contribute to an overall change in expansive growth rate, while other changes may not.

Biophysical equations describing the wall deformation rate (plastic and elastic deformation rates), net water uptake rate (water uptake rate minus transpiration rate), and rate of change of turgor pressure (that couples the biophysical processes of water uptake and wall deformation) have been previously derived and validated with experimental results from pressure probe experiments [1,2,3]. Previously, these biophysical equations have been referred to as the ‘augmented growth equations’. Equation (1) describes the relative rate of change in volume of the cell wall chamber, (d*V*_cw_/*V*d*t*) = *v*_cw_, as the sum of the relative rate of irreversible (plastic) deformation of the wall, *ϕ* (*P* − *P*_C_), and the relative rate of reversible (elastic) deformation of the wall, (1/*ε*) d*P*/d*t* (see Appendix A for definitions and description of individual variables and terms).

(1) $$v_{\mathrm{cw}}=\phi \;\left(P-P_{C}\right)+\left(\frac{1}{\varepsilon }\right)\;\frac{\;dP}{dt}$$

*Rate of change in volume of the cell wall chamber* = *plastic deformation rate + elastic deformation rate*

Consider the general case of a single walled cell that is partly exposed to the atmosphere and is transpiring. Then, Equation (2) describes the relative rate of change in water volume of the cell, (d*V*_w_/*V*d*t*) = *v*_w_, as the difference in the relative rate of water uptake, *L*_p_ *A/V* (Δ*π* − *P*), and relative rate of transpiration, *v*_T_.

(2) $$v_{w}=L_{p}\;\frac{A}{V}\left(\Delta \pi -P\right)-v_{T}$$

*Rate of change in water volume* = *water uptake rate* − *transpiration rate*

Recognizing that the relative rate of change in water volume and relative rate of change in volume of the cell wall chamber are essentially equal during expansive growth, i.e., *v*_w_ ≅ *v*_cw_, an equation for the turgor pressure behavior can be obtained. Equation (3) describes the rate of change of turgor pressure, *P*, in the cell.

(3) $$\;\frac{\;dP}{dt}=\varepsilon \;\left[\;L_{p}\;\frac{A}{V}\;\left(\Delta \pi -P\right)-\phi \;\left(P-P_{C}\right)-v_{T}\right]$$

*Rate of change of turgor pressure* = ε [*water uptake rate* − *plastic deformation rate of the wall* − *transpiration rate*]

These equations, Equations (1)–(3), are applicable to cells with walls that exhibit expansive growth, i.e., plant, fungal, and algal cells. It is noted that the turgor pressure is an important and explicit biophysical variable in each biophysical equation. The turgor pressure is a gage pressure and is defined as the difference in pressure inside, *P*_i_, and outside, *P*_o_, the plasma membrane; *P* = *P*_i_ − *P*_o_. The pressure probe was designed to directly measure and manipulate the turgor pressure in walled cells; see review [4] for details. Since its introduction, many pressure probe methods (experimental protocols employing the pressure probe) have been developed to measure the magnitude of the inclusive biophysical variables in Equations (1)–(3); *P*, *ϕ*, *ε*, *P*_C_, *L*_p_ and *v*_T_.

Here, it is shown how Equations (1)–(3) can be used to analyze and guide the use of pressure probe methods to determine the magnitudes of plastic and elastic wall deformation rates, water uptake rates, and transpiration rates before and after changes in environment, development, and mutations. Analyses using Equations (1)–(3) can assist in determining which of the inclusive biophysical variables (*P*, *ϕ*, *ε*, *P*_C_, *L*_p_, *π*_i_, and *v*_T_) should be determined with pressure probe methods and other relevant methods. Additionally, it is shown how Equations (1)–(3) can be used to interpret the experimental results. Because the magnitude of each biophysical variable (*P*, *ϕ*, *ε*, *P*_C_, *L*_p_, *π*, and *v*_T_) represents the magnitude of a related biological process involved in expansive growth, it is possible to identify the biological processes that are altered. The magnitude of these changes can be determined with the use of dimensionless Π parameters that have been obtained from dimensional analyses of the biophysical equations [5]. It is shown that the dimensionless numbers, obtained from relevant dimensionless Π parameters, can provide additional insights into expansive growth of plant, fungal, and algal cells [6,7,8].

In order to communicate the thinking and overall process of employing Equations (1)–(3) in concert with pressure probe methods, four cases that use the results of previously conducted experimental research are analyzed. Here, the analyses are conducted with the specific objective of determining which biophysical processes and biophysical variables in Equations (1)–(3) are altered. In some cases, it is possible to recommend additional experiments or modified pressure probe methods (i.e., experimental protocols employing the pressure probe) that can provide additional information to gain more insight. Each case follows the same format and includes the following:

(i) Introduction,
(ii) Objective,
(iii) Pre-analysis employing the biophysical equations,
(iv) Relevant experimental results and/or additional theoretical findings,
(v) Post-analysis employing the biophysical equations,
(vi) Discussion,
(vii) Conclusions, and
(viii) Recommendations.

The overall objective is to show how Equations (1)–(3) and pressure probe experiments can be employed in a symbiotic scheme to determine alterations in growth related biological processes. It is shown that it is not necessary to mathematically solve Equations (1)–(3) in order to use them for experimental design and analyses of experimental results.

## 2. Case 1: An Increase in Light Intensity Elicits an Increase in Growth Rate

### 2.1. Introduction

The sporangiophores of the fungus *Phycomyces blakesleeanus* are large aerial hyphae that grow vertically from the mycelium. The sporangiophores undergo five stages of development [9]. The stage IV sporangiophore is a single cylindrical cell (stalk) with a spherical sporangium on top. Elongation growth occurs in the cylindrical stalk that is approximately 15–45 mm in length and 0.1–0.18 mm in diameter. In constant uniform light and growth conditions, the stalk elongates at a nearly constant rate between 30 and 60 μm/min (basal growth rate). Interestingly, an increase in environmental light intensity elicits a transient increase in elongation growth rate that is nearly twice as large as the basal growth rate. The transient increase in elongation growth rate is termed the “light growth response” [9].

### 2.2. Objective

To determine which biophysical processes and biophysical variables are responsible for initiating and controlling the increase in elongation growth rate during the light growth response.

### 2.3. Pre-Analyses

An inspection of Equation (1) reveals that an increase in turgor pressure, *P*, will increase the plastic deformation rate of the wall (first term on the right hand side, RHS). The elastic deformation rate of the wall (second term on the RHS) increases when *P* increases, when d*P*/d*t* is positive. So, increasing *P* increases both the plastic and elastic deformation rate of the wall, and the elongation growth rate. On the other hand, an increase in elongation growth rate can be achieved without changing *P*, and instead increasing the magnitude of *ϕ* and/or decreasing the magnitude of *P*_C_. These changes in wall properties increase the plastic deformation rate of the wall. Because *P* remains constant, the elastic deformation rate is zero (d*P*/d*t* = 0). However, only plastic (irreversible and permanent) deformation of the wall is considered to be expansive growth. It is concluded from the pre-analyses that it is important to determine whether *P* increases during the light growth response.

### 2.4. Experimental Results

**Figure 1:**
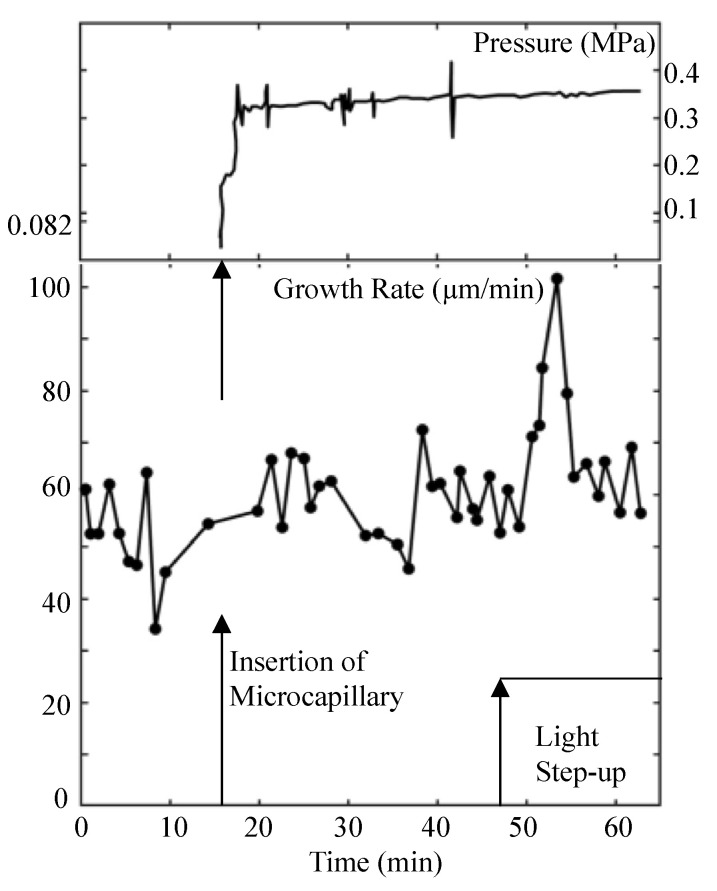
Turgor pressure behavior during the light growth response. Turgor pressure (top panel) and elongation growth rate (lower panel) are plotted against the same time scale. The top panel is the trace from the strip chart recorder showing the turgor pressure measured by a pressure probe. The vertical arrow on the time scale indicates the time when the sporangiophore was impaled to measure the turgor pressure. The vertical spikes on the pressure trace are produced by advancing and retracting the control rod of the pressure probe to assure the interface in the microcapillary is free and that the pressure inside the cell is being measured. The lower panel is the elongation growth rate as a function of time. The elongation growth rate was determined every minute after the experiment began. The elongation growth rate fluctuates around a mean value (~56 μm/min) until approximately 50 min when it increases to approximately 100 μm/min, then decreases again to its basal growth rate. The vertical arrows on the time scale indicate the time when the cell was impaled to measure the turgor pressure (~16 min) and when the step-up in light intensity was produced (~47 min). Typically, a three to four minute latency period precedes the transient increase in elongation rate of the light growth response (50–57 min). This figure is redrawn from Figure 1 in Ortega et al. [10].

Experiments were designed and conducted to measure the turgor pressure before and during the light growth response using a pressure probe [10]. The result of one such the experiment is presented in Figure 1.

It is noted that the turgor pressure remained constant during the transient increase in elongation growth rate (light growth response) that occurs between 50 and 60 min on the time scale.

### 2.5. Post-Analyses

Because the turgor pressure did not increase during the light growth response, it is deduced that a transient increase in wall deformation rate is solely responsible for the transient increase in elongation growth rate. Additionally, the constant turgor pressure decouples Equation (1) from Equations (2) and (3). This is important because this indicates that only Equation (1) is needed to determine the elongation growth rate during the light growth response. Additionally, the second term on the RHS of Equation (1) is zero (because *P* is constant and d*P*/d*t* = 0). Now it can be stated that a transient increase in plastic deformation rate of the wall (represented by the first term on the RHS) is solely responsible for the transient increase in elongation growth rate during the light growth response. Then, for the light growth response, Equations (1)–(3) can be replaced by a single governing equation, Equation (4). This equation was first derived by Lockhart [11].

(4) $$v=\phi \;\left(P-P_{C}\right)$$

An inspection of Equation (4) reveals that a transient increase in *ϕ* and/or a transient decrease in *P*_C_ must occur in order to produce the light growth response.

### 2.6. Discussion

It is shown that the transient increase in elongation growth rate of the light growth response is produced by changing the deformation properties of the wall to increase the plastic deformation rate, i.e., by increasing *ϕ* and/or decreasing *P*_C_. Furthermore, it is shown that the elongation growth rate is governed by a single equation, Equation (4). The sporangiophore of *P. blakesleeanus* exhibits another growth response to an environmental stimulus. If two microscope slides (parallel and 2 mm apart) are maneuvered to slide around the sporangiophore stalk without touching it, a transient increase in elongation growth rate is observed (avoidance growth response) [9]. The results of the iterative scheme used here for the light growth response predicts that the plastic deformation rate of the wall is regulated to elicit the avoidance growth response. In other words, it is predicted that *P* will not increase during the avoidance growth response. Experiments conducted with the pressure probe demonstrate that *P* is constant during the avoidance growth response [10]. It follows that Equation (4) governs the elongation growth rate during the avoidance growth response as well.

The results presented in this case highlight the importance of developing experimental methods to determine the magnitude of *ϕ* and *P*_C_. Subsequently, step-up in turgor pressure (*P* step-up) experiments were designed and shown to determine the magnitudes of *ϕ* and *P*_C_ in Equations (1) and (4) for stage IV sporangiophores of *P. blakesleeanus* [12]. Other step-up in *P* experiments were designed and shown to determine the magnitude of *ε* [13,14,15]. The theory of these pressure probe methods will be covered in more detail in the next section, Case 2.

### 2.7. Conclusions

It is concluded that *ϕ* and *P*_C_ change in magnitude in order to produce the light growth response. Theoretical research by Passioura and Fry [16] predict that increasing the rate of breaking load-bearing bonds between polymers in the wall increases the magnitude of *ϕ* and decreases the magnitude of *P*_C_. If this prediction is correct, the experimental and theoretical results presented here indicate that the main biological action needed to produce the light growth response is an increase in the rate of breaking load-bearing bonds between polymers in the wall. Other experimental research have identified pH [17] and some proteins (chitinase [18], chitin synthase [18], and expansins [19]) as candidates to regulate the rate of breaking load-bearing bonds in the cell walls of the sporangiophores of *P. blakesleeanus*.

Subsequent insight and support are obtained from dimensional analysis. Dimensional analysis was conducted on Equations (1)–(3) and dimensionless Π parameters were identified that could be used to determine the magnitude of each term in the dimensionless biophysical Equations (5)–(7). It was found that for growing sporangiophores of *P. blakesleeanus*, Equation (1) regulates the growth rate [8].

### 2.8. Recommendations

It is recommended that *P* step-up experiments be conducted to determine the magnitude of *ϕ* and *P*_C_ before and during the light and avoidance growth responses.

## 3. Case 2: A Comparison of the Biophysical Variables for Two Different Stages of Development

### 3.1. Introduction

The sporangiophores of *P. blakesleeanus* undergo five stages of development, two of which elongate at a different basal growth rates [9]. The basal growth rate for stage I sporangiophores is between 5 and 20 μm/min. The basal growth rate for stage IV sporangiophores is of between 30 and 60 μm/min.

### 3.2. Objective

To determine which biophysical processes and biophysical variables are responsible for the different basal growth rates of stage I and stage IV sporangiophores.

### 3.3. Pre-Analyses

Equation (1) shows that a change in *P* can simultaneously change both the plastic and elastic deformation rate of the wall, and the overall growth rate. A change in *P* requires an evaluation of Equations (1)–(3) and the biophysical variables, *ϕ*, *ε*, *P*_C_, *L*_p_, *π*_i_, and *v*_T_. If *P* is constant and the same magnitude, the difference in basal growth rate can be explained by changing the plastic deformation rate of the wall, see Equation (1). A change in *ϕ* and/or *P*_C_ can alter the wall’s plastic deformation rate and a change in *ε* can alter the wall’s elastic deformation rate when d*P*/d*t* is not zero. Similar to Case 1, it is thought that the turgor pressure must be measured in stage I and stage IV sporangiophores. Based on the results obtained for Case 1, it is also thought that the wall deformation rates may be important and perhaps responsible for the different basal growth rates. So, it is concluded that the wall properties (*ϕ*, *ε*, and *P*_C_), as well as the turgor pressure should be determined initially.

### 3.4. Experimental Results and Theoretical Findings

Step-up turgor pressure (*P* step-up) experiments were designed and conducted to determine the magnitudes of *ϕ*, *P*_C_, and *ε* in Equation (1) for stage IV sporangiophores of *P. blakesleeanus* [12]. The pressure probe experiments were designed to determine the magnitudes of *ϕ* and *P*_C_ by producing a small step-up in *P* with the pressure probe (Δ*P* ≈ 0.02 MPa). The elongation growth rate, before and after the *P* step-up, is measured in order to determine Δ*v*. It is shown that the magnitude of *ϕ* **before** the *P* step-up can be calculated with Equation (5) [12].

(5) $$\phi =\frac{\Delta v}{\Delta P}$$

Now because *v*, *ϕ*, and *P* are known and constant for the time interval before the *P* step-up, *P*_C_ can be calculated with Equation (6) [12].

(6) $$P_{C}=P-\frac{v}{\emptyset }$$

This *P* step-up method determines the magnitudes of *ϕ* and *P*_C_ for the time interval **before** the *P* step-up, and during steady growth rate.

A step-up in *P* is also used to determine the magnitude of *ε*. Equation (7) can be used to determine *ε* for growing, as well as nongrowing, cells because it was shown that the plastic wall deformation that occurs during a step change in *P* is very small and may be neglected [15].

(7) $$\varepsilon =l\;\frac{\Delta P}{\Delta l}$$

Experiments revealed that the Δ*l* immediately after a small Δ*P* of 0.02 MPa was almost undetectable for the sporangiophores [12], so a larger Δ*P* (Δ*P* ≈ 0.10 MPa) was used to determine *ε* [12,13,15,20].

After establishing the pressure probe methods using stage IV sporangiophores [12], the *P* step-up experiments were conducted on stage I sporangiophores of *P. blakesleeanus* [20,21]. The magnitudes for the biophysical variables *v*, *P*, *ϕ*, *P*_C_, and *ε* for stage I and stage IV sporangiophores are summarized in Table 1.

### 3.5. Post-Analyses

The experimental results presented in Table 1 show that the basal elongation growth rates of stage IV sporangiophores are larger than those of stage I sporangiophore. It is noted that the magnitude of *ϕ* increases with the growth rate while the magnitudes of *P*, *P*_C_, and *ε* decrease. Based on the reasoning in the pre-analyses, and because the turgor pressures are constant but significantly different for stage I and stage IV, it is concluded that the biophysical variables in Equation (2) must be determined. Experiments were conducted to determine the magnitude of *π*_I_ and *L*_p_ for stage I and stage IV [22]. Additional experiments were conducted to determine the magnitude of the transpiration rates in stage IV sporangiophores [13,23]. The transpiration rates for stage I sporangiophores were not determined. The magnitudes for the biophysical variables *π*_i_, *L*_p_, and *v*_T_ for stage I and stage IV sporangiophores are summarized in Table 2.

The measured values for the osmotic pressure in Table 2 for stage I and stage IV were not separated, so no distinction between the two can be obtained. However, it is noted that the magnitude of the hydraulic conductivity of the plasma membrane, *L*_p_, of stage I sporangiophores is larger than that for stage IV sporangiophores. Additionally, the transpiration rate was only determined for stage IV sporangiophores.

### 3.6. Discussion

The experimental results demonstrate that *ϕ* increases as the growth rate increases from stage I to stage IV. In contrast, *P*, *P*_C_, and *ε* decrease from stage I to stage IV, as does the driving force for plastic deformation rate of the wall, (*P − P_C_*) *A*_c_. The overall increase in plastic wall deformation rate is the product of the large increase in *ϕ* and the small decrease in (*P − P_C_*). The observed decrease in *ε* is supported by the findings of Proseus et al. [14], where it was found that the magnitude of *ε* decreases as the growth rate increases for the internode algal cells of *Chara corallina*.

Equations (2) and (3) can be used to explain the smaller *P* observed in stage IV compared to stage I. Because the elongation growth rate increases from stage I to stage IV, the rate of water uptake must increase to accommodate the increase in volumetric growth rate. An increase in osmotic water uptake rate can be achieved by decreasing *P*. Inspection of Equation (2) indicates that *v*_w_ will increase when *P* decreases, if *L*_p_, Δ*π*, and *v*_T_ remain constant. Although the behavior of Δ*π* was not measured, it is noted that *L*_p_ decreases for stage IV sporangiophores. Thus, a smaller *L*_p_ may require a larger decrease in turgor pressure to accommodate the increase in growth rate. So, the smaller magnitude of *P* for stage IV sporangiophores reflects the increase in growth rate and the smaller magnitude of *L*_p_. Support for this turgor pressure behavior can be obtained using Equation (3). Equation (3) shows that if the rate of plastic deformation of the wall increases, and if the rate of water uptake and transpiration remain constant, the rate of change of the turgor pressure, d*P*/d*t*, becomes negative. *P* will continue to decrease until the *net* water uptake rate (water uptake rate − transpiration rate) increases to accommodate the magnitude of the increased rate of plastic deformation of the wall.

The experimental results summarized in Table 1 demonstrate that the magnitude of *ϕ* increases with the growth rate while *P*_C_ decreases. This behavior of *ϕ* and *P*_C_ supports the theoretical findings of Passioura and Fry [16] where it is predicted that increasing the rate of breaking load-bearing bonds between polymers in the wall increases the magnitude of *ϕ* and decreases the magnitude of *P*_C_. Overall, the theoretical and experimental findings indicate that regulating the rate of breaking load-bearing bonds between polymers in the wall is a viable method to control plastic deformation rate of the wall and expansive growth rate of the sporangiophores of *P. blakesleeanus*. This finding may be extended to walls of other species of fungal, algal, and plant cells.

In Case 1, it was shown that Equation (1) governs the growth rate during the light growth response. Furthermore, the governing equation could be reduced to Equation (4) that describes the plastic deformation rate of the wall. Here, in Case 2, a more complicated picture emerges indicating biological control of both wall deformation rate (changing *ϕ*, *P*_C_, and *ε*) and water uptake rate (*P* and *L*_p_). So, in this case, one may ask, which biophysical process controls the elongation growth rate, net water uptake rate or wall deformation rate? A more formal method may be used to determine which biophysical process governs and controls the overall expansive growth rate. As mentioned, the rate of net water uptake and rate of wall deformation are interrelated and simultaneous. Overall, because these two biophysical processes occur in parallel and simultandously, the slowest of these two biophysical processes governs the rate of expansive growth. Previously, it was shown that the dimensionless forms of Equations (1)–(3) yield dimensionless coefficients, Π parameters, that represent the magnitude of each term in the dimensionless biophysical equations [5,6,7,8]. (A three-page review [7] may be helpful to understanding Π parameters for those who are not familiar with the subject.) One parameter, Π_wd_, was derived and shown to be the ratio of the magnitudes of Equations (1) and (2) [8]. The process of dimensional analysis provides the interpretation for the Π parameters as the ratio of two biophysical processes [5]. For Π_wd_, the first subscript, w, refers to the numerator (net water uptake rate) and the second subscript, d, refers to the denominator (deformation rate of the wall). In terms of other Π parameters and the biophysical variables in Equations (1)–(3), Π_wd_ is defined as follows.

$$\Pi_{\mathrm{wd}}=\left(\frac{\Pi_{\mathrm{wv}}-\Pi_{\mathrm{Tv}}}{\Pi_{\mathrm{pv}}+\Pi_{\mathrm{ev}}{\;}_{\;}{}^{\;}\;}\right)=\left(\frac{\frac{L\;\;P_{C}}{v}-\frac{v_{\mathrm{sT}}\;\;}{v\;}}{\frac{\;\phi \;P_{C}}{v}+\frac{\;P_{C}}{\varepsilon }\;}\right)=\frac{magnitude\;of\;net\;water\;uptake\;rate}{magnitude\;of\;wall\;deformation\;rate}$$

It was shown that when the dimensionless number calculated from Π_wd_ is greater than unity, the wall deformation rate governs the expansive growth rate, because the slowest process controls the overall growth rate [8]. The magnitude of Π_wd_ was calculated to be 236 for stage I, and 11 for stage IV [8]. These magnitudes indicate that the wall deformation rate and Equation (1) govern the elongation growth rate for both stage I and stage IV in normal conditions [8].

### 3.7. Conclusions

It is concluded that the difference in basal growth rate of stage I and stage IV sporangiophores is the result of the difference in the plastic deformation rates of the wall. The magnitude of the irreversible wall extensibility, *ϕ*, is predominately responsible for the larger basal growth rate of stage IV sporangiophores, but the smaller driving pressure, (*P* − *P*_C_), somewhat reduces the affect of the larger *ϕ*. At a molecular level, the difference in plastic wall deformation rates can be explained by the different rates of breaking load-bearing bonds in the walls of stage I and stage IV sporangiophores.

### 3.8. Recommendations

It is recommended that the osmotic pressures, for stage I and stage IV sporangiophores, be determined separately. Additionally, it is recommended that the transpiration rates of stage I sporangiophores be measured and compared to those of stage IV sporangiophores.

## 4. Case 3—A Decrease in Temperature Terminates Elongation Growth

### 4.1. Description

The internode of *C. corallina* is a large single algal cell that grows in length at approximately 0.2 μm/s. Young growing internodes are approximately 0.8 mm in diameter and 6 mm in length, and older growing internodes are approximately 1.2 mm in diameter and 22 mm in length. Single internodes are excised from the rest of the plant for experimentation and grow at slower rates (0.002–0.03 μm/s). The excised internodes have been used as a model system for plant growth [24]. Proseus et al. [14,24] employed the pressure probe to study the growth responses of excised internode cells to rapid change in turgor pressure and temperature. Proseus et al. [24] discovered that a decrease in temperature from 23 to 8 °C stopped elongation growth.

### 4.2. Objective

To determine which biophysical processes and biophysical variables are responsible for the termination of elongation growth after the decrease in temperature from 23 to 8 °C.

### 4.3. Pre-Analyses

An inspection of Equations (1)–(3) indicates that it is important to determine whether the turgor pressure changes after a decrease in temperature. It is recognized that the water uptake rate will change if the *P* is altered. If *P* increases or decreases, the experimental results must be analyzed in terms of Equations (1)–(3). If *P* remains constant, then the deformation properties of the wall are solely responsible for the change in elongation growth rate and its termination, and only Equation (1) needs to be evaluated.

### 4.4. Experimental Results

Experiments were conducted by Proseus et al. [24], where the pressure probe was employed to measure and manipulate the turgor pressure in excised internode cells of *C. corallina* before and after a decrease in temperature from 23 to 8 °C. It was discovered that the turgor pressure decreased after the decrease in temperature. Therefore, the magnitude and behavior of the biophysical variables in Equation (2), *v*, *P*, *π*_i_, and *L*_p_, were determined and reported. Because the internodes grow submerged in a water bath and the cells do not transpire, *v*_T_ = 0 in Equation (2). A summary of the results is presented in Table 3. It is noted that the initial elongation growth rate, *v*_o_, goes to zero after the temperature is decreased from 23 to 8 °C. The turgor pressure decreases to approximately 92% after the decrease in temperature. The decrease in *P* was explained by the decrease in magnitudes of osmotic pressure, (*π*_2_ = 0.95 *π*_o_), and hydraulic conductivity of the plasma membrane, (*L*_p2_ = 0.77 *L*_po_).

### 4.5. Post-Analyses

The results presented in Table 3 show that *v*, *P*, *π_i_*, and *L*_p_ all decrease after the temperature is lowered. It is noted that the driving force for water uptake, (Δ*π* − *P*) *A*, is slightly increased because both P and *π* decrease after the change in temperature, but P decreases slightly more; (Δ*π* − *P*) *A* = (0.95 *π_o_* − 0.92 *P_o_*) *A*. This together with the decrease in *L*_p_ indicates that the rate of water uptake rate is only slightly decreased after the decrease in temperature. Therefore, it is concluded that while the water uptake rate is slightly decreased, this finding alone cannot explain the termination of the elongation growth rate.

**Figure 2:**
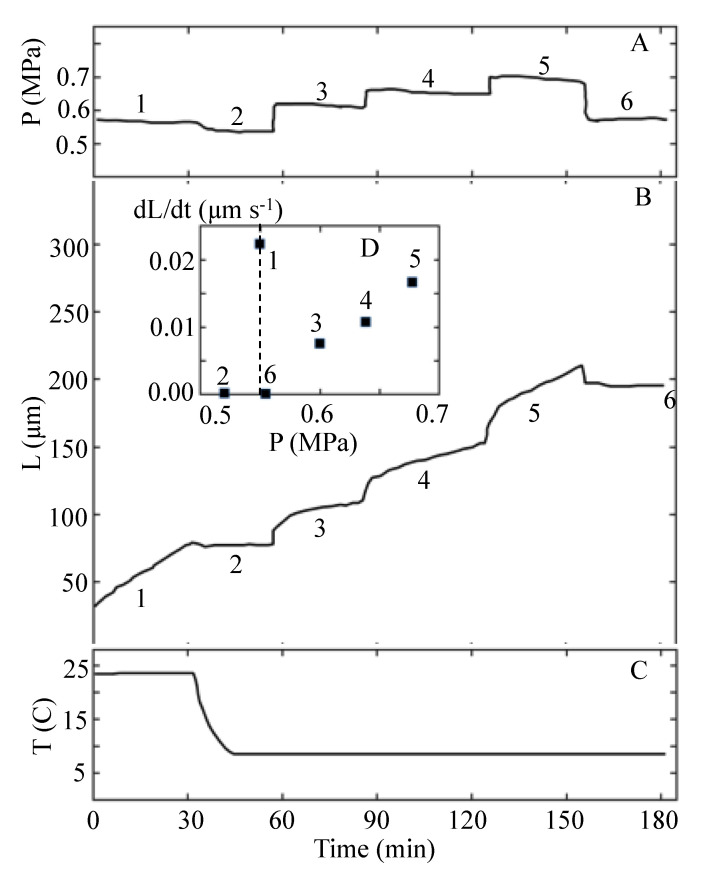
The turgor pressure, *P*, the length of the internode, *L*, and the temperature, *T*, are plotted against the same time scale. The top panel, **A**, shows the turgor pressure trace from the pressure probe. Note that six time intervals (1–6) of approximately 30 min duration are identified in Panels **A** and **B**. Panel **B** shows the length of the internode as a function of time after the start of the experiment. The inset, **D**, in Panel **B** shows the average elongation growth rate measured for each of the six time intervals. The bottom panel, **C**, shows the temperature as a function of time. Note that the temperature is sharply decreased from 23 to 8 °C during the second time interval. This figure is redrawn from Figure 12 in Proseus et al. [24].

Proseus et al. [24] did not report values for the biophysical variables in Equation (1). However, it is possible to determine values for *P*, *ϕ*, *ε*, and *P*_C_ from the results of the experiment that are reported in Figure 12 of Proseus et al. [24]. Figure 12 is redrawn and presented here as Figure 2.

The *P* steps-up produced with the pressure probe (after the temperature was lowered to 8 °C and toward the end of time intervals 2, 3, and 4) are used to determine the magnitude of *ϕ*, *P*_C_, and *ε* for the internode in cold conditions. The details of the calculations are presented in Appendix B. A summary of the calculated values for the biophysical variables for each time interval in Figure 2 is presented in Table 4. The magnitudes for *v* and *P* presented for the first time interval (*T*_1_ = 23 °C) are prior to the decrease in temperature. Values for *P*_C_, *ϕ*, and *ε* for the first time interval cannot be calculated because a *P* step-up was not conducted before the temperature was decreased. After the decrease in *T*, a *P* step-up was produced toward the end of each time interval. Therefore, *P*_C_, *ϕ*, and *ε* can be calculated using the data in Figure 2 together with Equations (5)–(7); see Appendix B. The calculated values for time intervals 2–4, when (*T* = 8 °K), are summarized in Table 4. It is noted that for interval 2, *v* = 0 and *P − P*_C_ = 0.

### 4.6. Discussion

The results presented in interval 2 of Table 4 reveal that both *v* and *P − P*_C_ decreases to zero when the temperature is lowered from 23 to 8 °C. This analyses show that the elongation growth rate is terminated because lowering the temperature from 23 to 8 °C decreased the turgor pressure to the magnitude of the critical turgor pressure so that the driving force for expansive growth is eliminated, i.e., (*P − P*_C_) *A*_c_ = 0. This conclusion is consistent with that of Proseus et al. [24], who did not employ Equations (1)–(3) for analyses, but used additional experimental results and reasoning. Interestingly, the results presented in intervals 2–4 demonstrate that *ϕ* is not zero. The values for *ε* at lower temperatures (8 °C) appear to be slightly smaller compared to those measured for internode cells growing at similar rates (0.007–0.016 μm/s) and at room temperature (23 °C); Proseus et al. [14], Figure 6.

### 4.7. Conclusions

Analyses using the biophysical equations and pressure probe experiments show that the elongation growth rate is terminated after the decrease in temperature from 23 to 8 °C because the turgor pressure decreases to the magnitude of the critical turgor pressure and eliminates the driving force for expansive growth. Based on additional experiments, Proseus et al. [24] reasoned that the magnitude of *P*_C_ was increased by lowering the temperature to 8 °C. The analyses with the biophysical equations conducted here, cannot confirm this because *P*_C_ could not be determine in the time interval before the temperature was decreased. If a *P* step-up were produced toward the end of interval 1, *P*_C_, *ϕ*, and *ε* could be determined for that time interval and compared to the respective values obtained subsequent to the decrease in temperature. Then, a direct comparison of the magnitude of *P*_C_ at 23 and 8 °C could confirm this conclusion and even determine the magnitude of the increase in *P*_C_. It appears that the remaining biophysical variables (*π*, *L*_p_, *ϕ*, and *ε*) are diminished in magnitude after the decrease in temperature, but are not zero. Therefore, small growth rates can be achieved by raising the turgor pressure above the magnitude of the critical turgor pressure.

### 4.8. Recommendations

The behavior of the biophysical variables in Equations (1)–(3) as a function of temperature would be very important to exploring the quantitative affects of changing temperature on expansive growth of plant cells. Therefore, it is recommended that pressure probe experiments, similar to those conducted for Figure 2 but with a *P* step-up toward the end of interval 1, be conducted and repeated for different temperatures above and below room temperature. If the experiments are repeated at each temperature enough times to determine statistical differences, then the analyses with the biophysical equations, as demonstrated here, can provide a quantitative relationship between the temperature and each of the biophysical variables, *v*, *P*, *L*_p_, *P*_C_, *ϕ*, and *ε* for the internode cells of *C. corallina*.

## 5. Case 4—Growth Mutants

### 5.1. Description

The stage IV sporangiophore of *P. blakesleeanus* grows toward a light source. This growth response to unilateral light is called the phototropic response [25]. Two mutant strains, C149 and C216, exhibit weak phototropic responses compared to wild type. They have been termed “stiff” mutants because of their inability to grow (bend) toward light [26,27,28]. Interestingly, the elongation (basal) growth rates of the stiff stage IV sporangiophores are similar to those of wild-type stage IV sporangiophores.

### 5.2. Objective

To determine which biophysical processes and biophysical variables are responsible for the diminished phototropic response in stiff mutant sporangiophores of C149 and C216 strains.

### 5.3. Pre-Analyses

Differential deformation rates on the distal and proximal sides of the sporangiophore stalk are required to produce bending during elongation growth. So, it was hypothesized that diminished differential deformation rates on the distal and proximal sides of the wall of the stalk must be responsible for the weak phototropic response. This was the reason the mutant strains were termed “stiff” mutants [25,26,27,28]. Because the deformation behavior of the wall is governed by Equation (1), initially the magnitudes of the biophysical variables in Equation (1) should be determined for the stiff mutants and compared to those of wild type.

### 5.4. Experimental Results

Step-up turgor pressure experiments employing the pressure probe were conducted on stage IV sporangiophores of mutant strains C149 and C216 [29]. The experimental protocol was identical to those conducted on wild-type stage I and stage IV sporangiophores in Case 2. The results are summarized in Table 5.

### 5.5. Post-Analyses

Comparing the magnitudes of the biophysical variables for wild-type and stiff mutant sporangiophores demonstrate that the basal growth rates, *v*, are very similar. However, it is noticed that *P* is larger and *P*_C_ is smaller for the stiff mutants compared to the wild type. This indicates that the driving force, (*P − P_C_*) *A*_c_, for elongation growth is larger for stiff mutants compared to wild type. In contrast, the magnitudes of *ϕ* for the stiff mutants are smaller than those of wild type. The magnitudes of *ε* for stiff mutant and wild-type stage IV sporangiophores are nearly the same.

The smaller magnitudes of *ϕ* for stiff mutants suggest that the plastic deformation rate of the wall is smaller. Π_pv_ can be used to determine the magnitude of the plastic deformation rate of the wall for the stiff mutants and wild-type stage IV sporangiophores [5,8].

$$\Pi_{\mathrm{pv}}=\left(\frac{\;\phi \;P_{C}}{v}\right)=\left(\frac{\;relative\;volumetric\;plastic\;\;deformation\;rate\;of\;the\;wall}{\;relative\;volumetric\;growth\;rate}\right)$$

A ratio, Π_pv_ (wild type)/Π_pv_ (mutant), can be used to determine the relative magnitude of the plastic deformation rate of the growing walls of wild-type and stiff mutant stage IV sporangiophores. These ratios are calculated for both C149 and C216 stiff mutants; see Appendix C for calculations.

For C149 mutants

$$\frac{\Pi_{\mathrm{pv}}\;\left(\mathrm{wild}\;\mathrm{type}\right)}{\Pi_{\mathrm{pv}}\;\left(C149\right)}=\frac{7.43}{1.02}=7.3$$

For C216 mutants

$$\frac{\Pi_{\mathrm{pv}}\;\left(\mathrm{wild}\;\mathrm{type}\right)}{\Pi_{\mathrm{pv}}\;\left(C216\right)}=\frac{7.43}{0.82}=9.1$$

These ratios demonstrate that the magnitudes of the walls’ plastic deformation rate of wild-type stage IV sporangiophores are seven to nine times larger than those of the stiff mutants.

Similarly, Π_ev_ can be used to determine the magnitude of the elastic deformation rate of the wall for the stiff mutants and wild-type stage IV sporangiophore [5,8].

$$\Pi_{\mathrm{ev}}=\left(\frac{\;P_{C}}{\varepsilon }\right)=\left(\frac{\;relative\;volumetric\;elastic\;\;deformation\;rate\;of\;the\;wall}{\;relative\;volumetric\;growth\;rate}\right)$$

The ratio, Π_ev_ (wild type)/Π_ev_ (mutant), can be used to determine the relative magnitude of the elastic deformation rate of the growing walls of wild-type and stiff mutant stage IV sporangiophores. These ratios are calculated for both C149 and C216 stiff mutants; see Appendix C for calculations.

For C149 mutants

$$\frac{\Pi_{\mathrm{ev}}\;\left(\mathrm{wild}\;\mathrm{type}\right)}{\Pi_{\mathrm{ev}}\;\left(C149\right)}=\frac{0.0043}{0.0027}=1.6$$

For C216 mutants

(8) $$\frac{\Pi_{\mathrm{ev}}\;\left(\mathrm{wild}\;\mathrm{type}\right)}{\Pi_{\mathrm{ev}}\;\left(C216\right)}=\frac{0.0043}{0.0025}=1.7$$

These ratios demonstrate that the magnitudes of the walls’ elastic deformation rate of wild-type stage IV sporangiophores are 1.6–1.7-fold larger than those of the stiff mutants.

### 5.6. Discussion

It is interesting that the elongation growth rates of wild-type and stiff mutants are nearly the same considering that *ϕ* is so much smaller for the stiff mutants compared to wild type. Inspection of the first term on the RHS of Equation (1) reveals that the larger magnitude of (*P − P_C_*) compensates for the smaller magnitude of *ϕ* for the stiff mutants and produces similar growth rates. The implications of the values of *ϕ*, *P_C_*, and *ε* on the magnitudes of plastic and elastic deformation rates can be determined with Π_pv_ and Π_ev_, respectively. The ratios of Π_pv_ parameters demonstrate that the plastic deformation rate of the growing stiff mutants are much smaller than those of wild type. The ratio of Π_ev_ parameters demonstrates that the elastic deformation rate of the growing stiff mutants are slightly smaller, but similar to those of wild type. Because the magnitudes of Π_pv_ for stiff mutant sporangiophores are so much smaller than those of wild type, it is suggested that the magnitude of differential plastic deformation rate of the wall on the distal and proximal sides of the mutant sporangiophores will be smaller than that of the wild-type sporangiophore. Additionally, this smaller differential will produce a smaller phototropic response.

The relative magnitudes of Π_pv_ and Π_ev_ for C149, C216, and wild-type sporangiophores have implications on the name “stiff” mutants. In mechanics, stiffness relates to the elastic behavior of a material. By comparing the magnitudes of Π_ev_, Munoz and Ortega [15] showed that the elastic deformation rate, or stiffness behavior, of C149 and C216 sporangiophores is similar to those of wild type. By comparing the magnitudes of Π_pv_, it is shown that the plastic deformation rate, or viscous behavior, of the walls of C149 and C216 sporangiophores is very much smaller (more viscous) compared to wild type. These walls exhibit considerable larger viscous behavior (smaller plastic deformation) than those of wild type. It was suggested that a more accurate name for C149 and C216 mutants might be “viscous” mutants [15].

### 5.7. Conclusions

It is concluded that the smaller plastic deformation rates of the growing walls of C149 and C216 sporangiophores represent a smaller capacity to produce differential plastic deformation rates on the distal and proximal sides of the cylindrical stalk of the sporangiophore. Therefore, a diminished phototropic response is produced after a unilateral light stimulus. The biophysical variable most responsible for the diminished phototropic response is *ϕ*, which is considerable smaller in the mutant strains. Additionally, it is suggested that a more accurate descriptive term for the C149 and C216 mutant strains is “viscous” mutants, instead of “stiff” mutants.

### 5.8. Recommendations

Because it was found that the turgor pressure is higher in the C149 and C216 stage IV sporangiophores compared to wild type, it is recommended that the biophysical variables in Equation (2) be measured, i.e., measure *L*_p_, *π*, and *v*_T_ for C149 and C216 stage IV sporangiophores.

## 6. Discussion

### 6.1. Overview

An iterative scheme is presented to study alterations in expansive growth rate of plant and fungal cells that are produced by changes in environment (Cases 1 and 3), development (Case 2), and mutations (Case 4). The scheme iterates between (i) analyses with established biophysical equations, Equations (1)–(3), and (ii) results obtained from pressure probe experiments. It is shown that it is not necessary to obtain mathematical solutions to the biophysical equations in order to use the iterative scheme. The scheme can determine which of the biophysical processes (water uptake, transpiration, plastic wall deformation, and elastic wall deformation) are altered and which altered biophysical process is predominately responsible for the change in expansive growth rate. In addition, the scheme can determined which of the biophysical variables (*P*, *ϕ*, *ε*, *P*_C_, *L*_p_, *π*_i_, and *v*_T_) are altered and which are predominately responsible for the change in expansive growth rate. The determination of altered biophysical variables can be useful in identifying which biological processes are affected; see the following section.

### 6.2. Biological Meaning of the Biophysical Variables (π_i_, L_p_, v_T_, φ, ε, and P_C_)

Examples of how changes in magnitude of individual biophysical variables represent changes in relevant biological processes follows. A change in the magnitude of *π*_i_ represents a change in the concentration, *c*_i_, of active solutes inside the plasma membrane of the cell. The van’t Hoff’s formula provides an explicit relationship between the two variables, *π*_i_ = *RT c*_i_, (where *R* is the ideal gas constant and *T* is absolute temperature). It is known that active solutes are products of cell metabolism, so they are produced inside the cell. Active solutes may also be transported into the cell from outside the plasma membrane by active transport. Therefore, the magnitude of the osmotic pressure is regulated by generation of active solutes inside the cell and active transport of active solutes from outside the cell. The van’t Hoff formula demonstrates that the osmotic pressure is a function of temperature. Temperature is an important environmental condition that changes throughout the day, and throughout the seasons of the year. Because temperature is always variable, it is important to know which biophysical variables are temperature dependent.

A change in the magnitude of *L*_p_ represents a change in transport of water through the plasma membrane by a pressure difference, i.e., Δ*π − P*. The plasma membrane is impermeable to water, but the presence of water channels (aquaporins) in the membrane promotes water conductivity. Opening and closing the water channels and increasing or reducing the number of water channels in the plasma membrane can regulate the conductivity of water through the plasma membrane and the magnitude of the *L*_p_. Experimental evidence indicates that the magnitude of *L*_p_ is a function of temperature [24].

The water lost from a walled cell to the surrounding air is defined as transpiration. *v*_T_ is the relative rate of change in water volume lost to the surrounding air by transpiration. The amount of water available for expansive growth is the difference in water uptake by osmosis and water lost by transpiration. So, for terrestrial plants, fungi, and algae undergoing expansive growth, it is advantageous to regulate transpiration rate. For higher plants, ferns, and lycophytes, transpiration rates can be regulated by opening and closing stoma. Transpiration rates can also be passively regulated by covering the wall surface exposed to the air with a waxy cuticle. However, often cuticles are absent from exposed surfaces of walls undergoing expansive growth. Plants and fungi can combat this lack of a cuticle during expansive growth by reducing the surface area where expansive growth occurs, i.e., limiting the growth to a growth zone. Growth zones are found in stems and roots of terrestrial plants and single-celled sporangiophores of fungi. Because evaporation of water is the main physical process in transpiration, transpiration is a function of temperature, relative humidity, and wind speed of the surrounding air, i.e., boundary layer thickness.

An increase in the magnitude of *ϕ* represents an increase in the rate of breaking load-bearing bonds in the wall. At a macroscopic level, an increase in plastic deformation rate of the wall and expansive growth rate are observed when *ϕ* increases. At a microscopic level, the regulation of *ϕ* appears to be different for different species of plant, fungal, and algal cells [18,19,30,31,32,33,34,35]. An increase in *ϕ* may be achieved by increasing the rate and/or number of bond-breaking enzymes or non-enzymatic proteins [18,19,30,31,32,33]. Additionally, a change in wall conditions (such as a change in pH) may increase the magnitude of *ϕ* [17,34]. The calcium pectate cycle may also regulate the magnitude of *ϕ* in the walls of algae [35]. Because the rate of chemical reactions catalyzed by enzymes, proteins, and other biological cycles are generally a function of temperature, it may be deduced that *ϕ* is a function of temperature.

An increase in the magnitude of *ε* will decrease the rate of stretching load-bearing bonds. At a macroscopic level, a decrease in elastic deformation rate of the wall is observed. The magnitude of *ε* may be increased by increasing the number of polymers added and incorporated into the wall. This increases the number of load-bearing bonds that share the overall load (stress) produced by *P* and reduces the stretching of each bond. Biologically, an increase in *ε* may reflect an increase in synthesis and incorporation of load-bearing polymers into the wall. More subtle behavior is observed because the magnitude of *ε* is weakly dependent on the magnitude of *ϕ*. For example, if the rate at which polymers are added and incorporated into the wall is constant, then increasing the rate of breaking load-bearing bonds, i.e., increasing *ϕ*, will reduce the number of load-bearing bonds in the wall and increase the load and stretching of the remaining unbroken load-bearing bonds. Macroscopically, this will be observed as a decrease in the magnitude of *ε*, and an increase in the elastic deformation rate of the wall. The described relationship between *ε* and *ϕ* is supported by the results presented here in Table 1, and in Figure 6 of Proseus et al. [14] where it is shown that the magnitude of *ε* decreases when the elongation growth rate increases, i.e., when *ϕ* increases. In most inert materials, the elastic modulus is a weak function of temperature. However, because of the relationship between *ε* and *ϕ*, it is deduced that *ε* is somewhat more dependent on temperature because *ϕ* is dependent on temperature.

*P*_C_ is defined as the magnitude of turgor pressure that must be exceeded before the rate of plastic deformation of the wall begins. At a molecular level, the magnitude of *P*_C_ represents the minimum stress in the wall that must be exceeded before the rate of bond-breaking between load-bearing polymers can cause polymers to continuously slide passed each other, separate, and generally relocate relative to each other. At a macroscopic level, plastic deformation rate of the wall is observed. It follows that the magnitude of *P*_C_ depends on the (i) number of load-bearing bonds in the wall, (ii) the average load on each bond, and (iii) the magnitude of the rate of bond-breaking of load-bearing bonds. If the bond-breaking rate of load-bearing bonds is constant, it is predicted that *P*_C_ will increase with an increase in the rate by which polymers are incorporated into the wall. This is because incorporating more polymers into the wall will increase the number of load-bearing bonds to share the overall load, and reduce the load on each bond. In contrast, if the rate of polymer incorporation into the wall is constant, it is predicted that the magnitude of *P*_C_ will decrease when the rate of bond-breaking of load-bearing bonds increases, i.e., *ϕ* increases. This is because increasing the rate of bond-breaking decreases the number of load-bearing bonds to share the overall load, and increases the load on each bond. The prediction that *P*_C_ decreases when *ϕ* increases, is supported by the experimental results presented here in Table 1 [21] and by the theoretical findings of Passioura and Fry [16]. Again, because there exists a relationship between *P*_C_ and *ϕ*, it is deduced that *P*_C_ is dependent on temperature.

### 6.3. Systematic Method for Analyses of Expansive Growth of Walled Cells

The plant or fungal cell may be considered as a single system that contains all the components needed to operate. From a system perspective, Equations (1)–(3) represent a set of *system equations* that govern its operational behavior during expansive growth. The two subsystems that regulate expansive growth rate are wall deformation rate and net water uptake rate. Each of these subsystems has a governing equation that controls its rate; Equation (1) and Equation (2), respectively. These two governing equations are interdependent and coupled by the turgor pressure. The turgor pressure behavior is described by Equation (3). This perspective can facilitate the employment of the iterative scheme demonstrated in Cases 1–4. Initially, it is always important to state the objective in explicit terms. Pre-analyses to determine which subsystem may be affected, and which can evoke the observed response, is helpful in deciding which pressure probe method(s) to use initially. After the pressure probe experiments are conducted, of course it is important to analyze the results. However, analyzing the results from the perspective of Equations (1)–(3) can provide a broader and more relevant perspective of the affected subsystems and their inclusive biophysical variables. Overall, the iterative scheme presented here provides a systematic approach to investigating how and why the expansive growth rate of walled cells is altered by changes in the environment, development, and mutations.

### 6.4. Global and Local Equations

Expansive growth rates of individual walled cells are analyzed in Cases 1–4. It is important to recognize that the biophysical equations are *global*. This means that the locations the biophysical processes are not considered, and the magnitude of the biophysical variables are considered to be an average value for the whole cell. In other words, the cell system is considered to be a “lumped” system and its properties are considered homogeneous throughout the system. The global biophysical equations, Equations (1)–(3), govern the lumped system’s behavior during expansive growth. It follows that the magnitude of the inclusive biophysical variables are average values for the lumped system. Typically, this is not a problem for some biophysical variables such as the turgor pressure, because it is uniformly distributed throughout the vacuole and cell sap, and is isotropic in its action. However, other biophysical variables that describe the deformation rate of the wall can be very different along the surface of the cell wall chamber. This is especially true for cells that exhibit tip growth (where all the expansive growth occurs in a short region on the wall chamber, i.e., the growth zone) and cells that respond to environmental stimuli or undergoing morphogenesis (where some parts of the wall chamber undergo plastic deformation at a faster rate). It follows that Equation (1) cannot be used to investigate the differential deformation rate at different parts of a single wall chamber.

*Local* biophysical equations are required to study differential deformation rates at various locations on the wall chamber. Out of necessity, local equations are more complicated than global equations. Some local biophysical equations for wall deformation rate were reviewed by Ortega and Welch [36]. It is noticed that most of the constitutive (stress–strain) equations for the deformation behavior of the wall are described by continuum equations. Furthermore, some constitutive equations do not account for elastic deformation. A shortcoming of continuum equations is that the underlying molecular structure of the wall and dynamic molecular interactions that result in plastic and elastic deformation are not explicit. Recently, some progress as been achieved in addressing these concerns with the development of a statistical model for the wall during expansive growth [37]. In the statistical model, the dynamics of transient tether-microfibril networks are used to simulate the wall deformation response to the stress imposed by turgor pressure. The statistical model accurately described the global dynamics of the wall during (i) steady growth, (ii) stress relaxation, and (iii) response to a step-up in turgor pressure for fungal sporangiophores (*P. blakesleeanus*), algal internode cells (*C. corallina*), and plant tissue in the growth zone of pea (*Pisum sativus* L.). Additionally, the statistical model provides a molecular interpretation of the dimensionless time constant for wall stress relaxation, Π_pe_, that compliments the macroscopic interpretation.

While global equations cannot describe differential expansive growth rates on the wall chamber, they provide important advantages. First, they are mathematically simpler than local equations. Second, they are more intuitive and easier to interpret compare to local equations. Third, they are easier to validate with experiments. Importantly, the global equations, Equations (1)–(3), have been validated with results obtained from pressure probe experiments [1,2,3]. Therefore, these equations are most accurate when they are employed in combination with pressure probe experiments. Some other sets of *global biophysical equations* that can be used in combination with pressure probe experiments are presented in Appendix D.

### 6.5. Analyses in Plant Tissue

Another important advantage of global equations is that they can be extended to growing plant tissue. Many plants exhibit expansive growth in a specific region of the stem or root (growth zone). The growing plant tissue in the growth zone is composed of expanding walled cells that are very similar to each other. Then, conceptually, theoretically, and as a first approximation, the whole growth zone can be considered to be a “lumped” system with homogeneous (averaged) biophysical properties throughout the system. The global biophysical equations presented here can be applied to the lumped system of the growth zone. Cosgrove [38] successfully employed Equation (1) to quantitatively explain “in vivo stress relaxation” in the growth zone of the stem of *Pisum sativus* L. It was shown that the same biophysical variables (*ϕ* and *ε*) in Equation (1) governed the rate of both stress relaxation and expansive growth of pea stem tissue. This finding is supported by analyses using the Π_pe_ parameter [6]. The results provide strong support for considering the growth zone as a single lumped system, because the magnitude of *ϕ* determined with stress relaxation experiments was shown to be the same as the magnitude of *ϕ* determined in elongation growth of the growth zone [6,39].

Theoretically treating the growth zone as a lumped system introduces a concern. Plant tissue uses their cell walls to transport water, nutrients, and solutes to all the inclusive cells. Cell walls and xylem are part of the apoplasm pathway. During expansive growth, the pressure within the apoplasm is often different from atmosphere pressure. Therefore, Equations (1)–(3) were extended to describe the wall deformation, water uptake, and turgor pressure, *P*_I_, when the pressure within the apoplast, *P*_A_, are higher or lower than atmospheric pressure [3]; also see Appendix D, Equations (A7)–(A9). It is seen that this set of global equations are more complicated than Equations (1)–(3). Some solutions to Equations (A7)–(A9) are obtained that are useful in understanding the affect of changing pressure in the apoplasm to plant growth [3]. In addition, some phenomena observed in plant growth behavior can be explained by solutions to Equations (A7)–(A9) [3]. Importantly, there are experimental tricks that allow the use of Equations (1)–(3) instead of Equations (A7)–(A9). It is noted that Equations (1)–(3) are recovered from Equations (A7)–(A9) when *P*_A_ = 0 (then *P*_I_ = *P*). Therefore, if the growing tissue is excised from the plant for experimentation, the pressure within the apoplasm comes to equilibrium with the atmospheric pressure, and Equations (1)–(3) can be used instead of Equations (A7)–(A9). In fact, incised tissue was used for the stress relaxation experiments in pea stem tissue [38].

The pressure probe methods used to measure *P* in cells of plant tissue are the same as those used for single-celled organisms [4,22]. However, *P* is measured in many cells distributed throughout the plant tissue and their magnitudes are averaged. The hydraulic conductance, *L*, may be estimated from tissue swelling experiments [38]. The osmotic pressure, *π*_i_, can be measured with a freezing-point osmometer, sampling cell sap from different regions of the growth zone or from expressed cell sap from growing tissue [39]. The in vivo stress relaxation method [38] can provide average magnitudes for *ϕ* and *P*_C_, if the average magnitude for *ε* is known. The average *ε* may be estimated from tissue hyration experiments [38] or transpiration experiments [39]. Additionally, the transpiration term, *v*_T_, can be eliminated experimentally by maintaining 100% relative humidity in an experimental chamber for both single cells [12] and plant tissue [38]. When measuring transpiration is important, measuring the change in weight of the growing tissue and growing cells with a very sensitive scale has been employed [40]. Some pressure probe methods are available to measure transpiration rates for single individual cells [13,23].

### 6.6. Analyses with Dimensionless Numbers

As shown in Cases 2 and 4, analyses with dimensionless numbers can provide additional insight into the experimental results. The Π_wd_ parameter was use to compare the magnitudes of different biophysical processes (net water uptake rate and wall deformation rate) for two stages of development (Case 2). It was found that the wall deformation rate was much smaller than the net water uptake rate for both stage I and stage IV sporangiophores. In Case 4, Π_pv_ and Π_ev_ parameters were used to compare the magnitudes of the same biophysical processes (plastic deformation rates and elastic deformation rates, respectively) for wild type and mutant cells of the same species. It was found that the plastic deformation rates of walls of wild-type sporangiophores are 7–9-fold larger than those of stiff mutant sporangiophores. However, the elastic wall deformation rates were nearly the same for wild type and mutant sporangiophores.

Previously, another dimensionless parameter, Π_pe_ = (*εϕ*/*v*), was shown to be the dimensionless ‘time constant’ for in vivo wall stress relaxation [6]. Analyses using Π_pe_ supports the hypothesis that wall stress relaxation is fundamental to expansive growth [6,41]. Experimentally determined values of Π_pe_ for plant, algal, and fungal cells demonstrate that their magnitudes are generally large and different [6]. Importantly, it appears that the magnitude of Π_pe_ is constant for a single species of growing walled cell or plant tissue. Furthermore it was shown that when the magnitude of Π_pe_ is experimentally determined for growing plant tissue (*Pisum sativus* L.), internode algal cells (*C. corallina*), and fungal sporangiophores (*P. blakesleeanus*), it is possible to accurately calculate the steady elongation growth rate without using Equations (1)–(3), even when the growth conditions change and the stage of development changes [6]. This finding provides a simple mathematical relationship between the magnitude of the growth rate, *v*, and the magnitudes of *ϕ* and *ε*, when Π_pe_ is measured experimentally with stress relaxation experiments. So, future research may show that when Π_pe_ is determined experimentally for any species of plant, fungi, or algae, the growth rate can be calculated with the simple equation, *v* = *εϕ*/Π_pe_, and changes in *v* can be determined when *ϕ* and *ε* change (but Π_pe_ remains constant).

More recently, all the Π parameters shown in Appendix D were determine and compared for single sporangiophore cells of *P. blakesleeanus*, single internode algal cells of *C. corallina*, and growing stem tissue of pea, *Pisum sativum* L. [8]. It was found for all three species that the wall’s plastic deformation rate is much larger than the elastic deformation rate. Additionally, it is found that the magnitude of Π_wd_ is approximately ten or greater for all three species, implying that the wall deformation rate regulates the expansive growth rate in all three species during normal growth conditions.

It is noteworthy that these dimensionless parameters have other uses that should be explored in future work concerning the affects of environmental changes on expansive growth of plants, algae, and fungi. Π parameters can (i) explicitly identify natural relationships between relevant variables, (ii) reduce the number of graphs needed to correlate data, (iii) be used to conduct ‘scale’ analyses, and (iv) be used to establish ‘similarity’ between processes [7]. For example, the principle of similarity can be used to help determine which bond-breaking enzymes, non-enzymatic proteins, and/or changes in wall properties create changes in the in vitro wall that are ‘similar’ to those in the in vivo wall, i.e., changes that produce ‘similarity’ deformation of the in vitro wall and in vivo wall [6].

## 7. Conclusions

It is shown that an iterative scheme (iterating between analyses employing biophysical equations and results obtained from pressure probe experiments) can determine the biophysical processes and biophysical variables that are altered after changes in environment, development, and mutations. Additionally, it is shown that this scheme does not require getting mathematical solutions to the biophysical equations. Analyses employing dimensionless Π parameters are used to determine the relative magnitude of the changes in biophysical processes and can help elucidate which biophysical variables are most responsible for changes in expansive growth rate. While the scheme was demonstrated for single fungal and algal cells, it is possible to extend the scheme to investigate growing plant tissue that have relatively homogeneous cell types, such as growth zones of plant stems and roots.

## Figures and Tables

**Table 1:** The magnitudes of the biophysical variables (*v*, *P*, *P*_C_, *ϕ*, and *ε*) obtained with the *P* step-up experiments for two stages of sporangiophore development, stage I and stage IV [12,20,21].

Variable (Units)	*P. blakesleeanus* (Stage I) Mean ± SE (*n*)	*P. blakesleeanus* (Stage IV) Mean ± SE (*n*)
*v* (h^−1^)	0.021 ± 0.002 (17)	0.068 ± 0.006 (20)
*P* (MPa)	0.48 ± 0.02 (17)	0.32 ± 0.01 (20)
*P*_C_ (MPa)	0.40 ± 0.03 (17)	0.26 ± 0.01 (20)
*P − P_C_* (MPa)	0.08 ± 0.01 (17)	0.05 ± 0.01 (20)
*ϕ* (h^−1^ MPa^−1^)	0.35 ± 0.06 (17)	2.00 ± 0.33 (20)
*ε* (MPa)	68.9 ± 5.6 (27)	60.9 ± 5.1 (27)

**Table 2:** The magnitudes of *π*, *L*_p_, and *v*_T_ were experimentally determined for two stages of development, stage I and stage IV [13,22,23]. The values for *π*_i_ were not measured separately for stage I and stage IV sporangiophores (*). Additionally, *v*_T_ for stage I sporangiophore have not been determined.

Variable (Units)	*P. blakesleeanus* (Stage I) Mean ± SE (*n*)	*P. blakesleeanus* (Stage IV) Mean ± SE (*n*)
*π*_i_ (MPa)	1.15 ± 0.05 (16) *	1.15 ± 0.05 (16) *
*L*_p_ (cm s^−1^ bar^−1^)	6.88 ± 0.5 × 10^−6^ (47)	1.96 ± 0.5 × 10^−6^ (42)
*v*_T_ (h^−1^)	----	0.12

**Table 3:** Measured values for the biophysical variables (*v*, *P*, *π*_i_, and *L*_p_) in Equation (2), as reported by Proseus et al. [24]. The initial values are denoted by the subscript (o).

Variable (Units)	*T*_1_ = 296 °K (23 °C)	*T*_2_ = 281 °K (8 °C)
*v* (h^−1^)	*v*_1_ *= v*_o_	*v*_2_*=* 0
*P* (MPa)	*P*_1_ = *P*_o_	*P*_2_ ≈ (0.92) *P*_o_
*π*_i_ (MPa)	*π*_1_ = *π*_o_	*π*_2_ = (*T*_2_/*T*_1_) *π*_o_
*L*_p_ (h^−1^ MPa^−1^)	*L*_p1_ = *L*_po_	*L*_p2_ = (0.77) *L*_po_

**Table 4:** Calculated magnitudes of the biophysical variables (*v*, *P*, *P*_C_, *P − P*_C_, *ϕ*, and *ε*) for each time intervals in Figure 2. Details of the calculations are presented in Appendix B.

Variable (Units)	*T*_1_ = 23 °C Interval 1	*T*_2_ = 8 °C Interval 2	*T*_3_ = 8 °C Interval 3	*T*_4_ = 8 °C Interval 4
*v* (h^−1^)	0.013	0.0	0.004	0.006
*P* (MPa)	0.57	0.52	0.60	0.65
*P*_C_ (MPa)	----	0.52	0.50	0.59
*P – P*_C_ (MPa)	----	0.0	0.10	0.06
*ϕ* (h^−1^ MPa^−1^)	----	0.05	0.04	0.10
*ε* (MPa)	----	41.0	31.0	37.0

**Table 5:** The magnitudes of the biophysical variables (*v*, *P*, *P*_C_, *P* − *P*_C_, *ϕ*, and *ε*) for stage IV stiff mutant sporangiophores (C216 and C149) are summarized and compared with those of stage IV wild-type sporangiophores [29]. The magnitudes of *ε* for wild type (*) are obtained from [20] and those of C216 and C149 (**) are obtained from [15].

Variable (Units)	Wild Type Mean ± SE (*n*)	C216 Mean ± SE (*n*)	C149 Mean ± SE (*n*)
*v* (h^−1^)	0.07 ± 0.01 (20)	0.07 ± 0.01 (18)	0.06 ± 0.01 (8)
*P* (MPa)	0.32 ± 0.01 (20)	0.40 ± 0.01 (18)	0.41 ± 0.02 (8)
*P*_C_ (MPa)	0.26 ± 0.01 (20)	0.13 ± 0.05 (18)	0.18 ± 0.08 (8)
*P − P*_C_ (MPa)	0.05 ± 0.01 (20)	0.27± 0.05 (18)	0.23 ± 0.06 (8)
*ϕ* (h^−1^ MPa^−1^)	2.00 ± 0.33 (20)	0.44 ± 0.08 (18)	0.34 ± 0.06 (8)
*ε* (MPa)	60.9 ± 5.1 (27) *	52.6 ± 4.4 (25) **	67.7 ± 7.3 (18) **

## Data Availability

Not applicable.

## Appendix A. Definitions and Description of Individual Variables and Terms

A= *area of the plasma membrane*
*A*_c_ = *cross-section area of a cylindrical cell*
$L_{P}=hydraulic\;conductivity\;$*of* *the plasma membrane*
$L=\left(\frac{L_{p\;}A}{\;V}\right)=relative\;hydraulic\;conductance\;of\;the\;plasma\;membrane$
$P=turgor\;pressure\;\left(gage\;pressure\;relative\;to\;the\;atmosphere\right)$
$P_{A}=pressure\;in\;apoplast,\;i.e.,\;cell\;wall\;\left(gage\;pressure\;relative\;to\;the\;atmosphere\right)$
$P_{C}=critical\;turgor\;pressure\;\left(pressure\;to\;be\;exceeded\;before\;plastic\;extension\;begins\right)\;$
t= *time*
V= *volume*
$V_{\mathrm{cw}}=volume\;$*of* *the cell wall chamber*
$V_{w}=volume\;$*of* *water in the cell*
$V_{T}=volume\;$*of* *water lost through transpiration*
$v_{\;}=\left(\frac{dV_{\;}}{\;\;V_{\;}\;dt{\;}_{\;}}\right)=relative\;rate\;of\;change\;in\;volume\;of\;the\;cell$
$v_{\mathrm{cw}}=\left(\frac{dV_{\mathrm{cw}}}{\;V\;dt{\;}_{\;}}\right)=relative\;rate\;of\;change\;in\;volume\;of\;the\;cell\;wall\;chamber$
$v_{T}=\left(\frac{dV_{T}}{\;V\;dt{\;}_{\;}}\right)=relative\;rate\;of\;change\;in\;water\;volume\;lost\;via\;transpiration$
$v_{w}=\left(\frac{dV_{w}}{\;V\;dt{\;}_{\;}}\right)=relative\;rate\;of\;change\;in\;water\;volume\;in\;the\;cell$
ε=volumetric elastic modulus of the cell wall
ϕ=relative irreversible extensibility of the cell wall
π=osmotic pressure
$\pi_{i}=internal\;osmotic\;pressure\;or\;osmotic\;pressure\;of\;the\;cell\;sap$
Δπ=osmotic pressure difference
Π=dimensionless number or nondimensional number
$L\;\left(\Delta \pi -P\right)=relative\;volumetric\;rate\;of\;water\;uptake$
$\phi \;\left(P-P_{C}\right)=relative\;volumetric\;plastic\;deformation\;rate\;of\;the\;cell\;wall$
$\frac{dP_{\;}}{\;\varepsilon {\;}_{\;}dt{\;}_{\;}}=relative\;volumetric\;elastic\;deformation\;rate\;of\;the\;cell\;wall$

## Appendix B. Calculations

Calculations for the values in Table 4 are conducted here. The data for the following calculations were estimated from an enlarged Figure 12 (400×) of a downloaded reprint of Proseus et al., 2000 [24]. The analyses employ Equations (5)–(7):

### Appendix B.1. Values for Interval 1 (T = 23 °C)

Values are taken directly from the legend of Figure 12 from Proseus et al. [24].

$$v_{1}=\left(\frac{1}{l}\right)\;\frac{\;dl}{dt}=\frac{0.022\;\mu m/s}{6000\;\mu m}\;\;\left(3600\frac{s}{h}\;\right)=\frac{0.013}{h}$$

$$P_{1}=0.57\;\mathrm{MPa}$$

### Appendix B.2. Values for Interval 2 (T = 8 °C)

$$v_{2}=\left(\frac{1}{l}\right)\;\frac{\;dl}{dt}=\frac{0.0\;\mu m/s}{6000\;\mu m}\;\;\left(3600\frac{s}{h}\;\right)=\frac{0.0}{h}$$

$$v_{3}=\left(\frac{1}{l}\right)\;\frac{\;dl}{dt}=\frac{0.0072\;\mu m/s}{6100\;\mu m}\left(3600\frac{s}{h}\;\right)=\frac{0.0043}{h}\;$$

$$P_{2}=0.52\;\mathrm{MPa}\;$$

$$P_{3}=0.60\;\mathrm{MPa}\;$$

$$\phi_{2}=\frac{\Delta v_{2}}{\Delta P_{2}}=\left(\frac{v_{3}-v_{2}}{P_{3}-P_{2}\;}\right)=\left(\frac{\frac{0.0043}{h}-\frac{0.0}{h}}{0.60\;\mathrm{MPa}-0.52\;\mathrm{MPa}}\right)=\frac{0.054}{h\;\mathrm{MPa}}\;$$

$$P_{C2}=P_{2}-\frac{v_{2}}{\phi_{2}}=0.52\;\mathrm{MPa}-\left(\frac{\frac{0.0}{h}}{\frac{0.053}{h\;\mathrm{MPa}}\;}\right)=0.52\;\mathrm{MPa}\;$$

$$\varepsilon_{2}=l_{2}\;\frac{\Delta P_{2}}{\Delta l_{2}}=l_{2}\left(\frac{P_{3}-P_{2}}{\Delta l_{2}\;}\right)=6000\;\mu m\left(\frac{0.60\;\mathrm{MPa}-0.52\;\mathrm{MPa}}{11.7\;\mu m}\right)=41.0\;\mathrm{MPa}$$

### Appendix B.3. Values for Interval 3 (T = 8 °C)

$$v_{3}=\left(\frac{1}{l}\right)\;\frac{\;dl}{dt}=\frac{0.0072\;\mu m/s}{6100\;\mu m}\;\;\left(3600\frac{s}{h}\;\right)=\frac{0.004}{h}$$

$$v_{4}=\left(\frac{1}{l}\right)\;\frac{\;dl}{dt}=\frac{0.0106\;\mu m/s}{6130\;\mu m}\left(3600\frac{s}{h}\;\right)=\frac{0.006}{h}$$

$$P_{3}=0.60\;\mathrm{MPa}$$

$$P_{4}=0.65\;\mathrm{MPa}$$

$$\phi_{3}=\frac{\Delta v_{3}}{\Delta P_{3}}=\left(\frac{v_{4}-v_{3}}{P_{4}-P_{3}\;}\right)=\left(\frac{\frac{0.006}{h}-\frac{0.004}{h}}{0.65\;\mathrm{MPa}-0.60\;\mathrm{MPa}}\right)=\frac{0.04}{h\;\mathrm{MPa}}$$

$$P_{C3}=P_{3}-\frac{v_{3}}{\phi_{3}}=0.60\;\mathrm{MPa}-\left(\frac{\frac{0.004}{h}\;}{\frac{0.04}{h\;\mathrm{MPa}}\;}\right)=0.5\;\mathrm{MPa}$$

$$\varepsilon_{3}=l_{3}\;\frac{\Delta P_{3}}{\Delta l_{3}}=l_{3}\left(\frac{P_{4}-P_{3}}{\;\Delta l_{3}}\right)=6100\;\mu m\left(\frac{0.65\;\mathrm{MPa}-0.60\;\mathrm{MPa}}{10.0\;\mu m}\right)=30.5\;\mathrm{MPa}$$

### Appendix B.4. Values for Interval 4 (T = 8 °C)

$$v_{4}=\left(\frac{1}{l}\right)\;\frac{\;dl}{dt}=\frac{0.0106\;\mu m/s}{6130\;\mu m}\left(3600\frac{s}{h}\;\right)=\frac{0.006}{h}$$

$$v_{5}=\left(\frac{1}{l}\right)\;\frac{\;dl}{dt}=\frac{0.0164\;\mu m/s}{6180\;\mu m}\left(3600\frac{s}{h}\;\right)=\frac{0.010}{h}$$

$$P_{4}=0.65\;\mathrm{MPa}$$

$$P_{5}=0.69\;\mathrm{MPa}$$

$$\phi_{4}=\frac{\Delta v_{4}}{\Delta P_{4}}=\left(\frac{v_{5}-v_{4}}{P_{5}-P_{4}\;}\right)=\left(\frac{\frac{0.010}{h}-\frac{0.006}{h}}{0.69\;\mathrm{MPa}-0.65\;\mathrm{MPa}}\right)=\frac{0.10}{h\;\mathrm{MPa}}$$

$$P_{C4}=P_{4}-\frac{v_{4}}{\phi_{4}}=0.65\;\mathrm{MPa}-\left(\frac{\frac{0.006}{h}}{\frac{0.10}{h\;\mathrm{MPa}}\;}\right)=0.59\;\mathrm{MPa}$$

$$\varepsilon_{4}=l_{4}\;\frac{\Delta P_{4}}{\Delta l_{4}}=l_{4}\left(\frac{P_{5}-P_{4}}{\;\Delta l_{4}}\right)=6130\;\mu m\left(\frac{0.69\;\mathrm{MPa}-0.65\;\mathrm{MPa}}{6.7\;\mu m}\right)=36.6\;\mathrm{MPa}$$

## Appendix C. Calculations of Πpv and Πev for Wild-Type, C149, and C216 Stage IV Sporangiophores

The values for the calculations are taken from Table 5. The process of dimensional analysis provides the interpretation for the dimensionless Π parameter as the ratio of two biophysical processes [5]. Here, subscripts are used to identify the biophysical processes involved. For example, he first subscript, p, of the Π_pv_ parameter refers to the numerator (relative plastic deformation of the wall) and the second subscript, *v*, refers to the denominator (relative volumetric growth rate).

$$\Pi_{\mathrm{pv}}\left(\mathrm{wild}\;\mathrm{type}\right)=\left(\frac{\;\phi \;P_{C}}{v}\right)=\left(\frac{\;\left(\frac{2.0}{h\;\mathrm{MPa}}\right)\left(0.26\;\mathrm{MPa}\right)}{\frac{0.07}{h}}\right)=7.43$$

$$\Pi_{\mathrm{pv}}\left(C149\right)=\left(\frac{\;\phi \;P_{C}}{v}\right)=\left(\frac{\;\left(\frac{0.34}{h\;\mathrm{MPa}}\right)\left(0.18\;\mathrm{MPa}\right)}{\frac{0.06}{h}}\right)=1.02$$

$$\Pi_{\mathrm{pv}}\left(C216\right)=\left(\frac{\;\phi \;P_{C}}{v}\right)=\left(\frac{\;\left(\frac{0.44}{h\;\mathrm{MPa}}\right)\left(0.13\;\mathrm{MPa}\right)}{\frac{0.07}{h}}\right)=0.82$$

$$\Pi_{\mathrm{ev}}\left(\mathrm{wild}\;\mathrm{type}\right)=\left(\frac{\;P_{C}}{\varepsilon }\right)=\left(\frac{\;0.26\;\mathrm{MPa}}{60.9\;\mathrm{MPa}}\right)=0.0043$$

$$\Pi_{\mathrm{ev}}\left(C149\right)=\left(\frac{\;P_{C}}{\varepsilon }\right)=\left(\frac{\;0.18\;\mathrm{MPa}}{67.7\;\mathrm{MPa}}\right)=0.0027$$

$$\Pi_{\mathrm{ev}}\left(C216\right)=\left(\frac{\;P_{C}}{\varepsilon }\right)=\left(\frac{\;0.13\;\mathrm{MPa}}{52.6\;\mathrm{MPa}}\right)=0.0025$$

## Appendix D. Additional Sets of *Global Biophysical Equations* That Can be Used with Pressure Probe Experiments

### Appendix D.1. Set of Global Biophysical Equations, When P = Constant and v_T_ Is Zero

(A1)
$$v_{\mathrm{cw}}=\phi \;\left(P-P_{C}\right)$$

*Rate of change in cell wall chamber volume* = *plastic deformation rate*

(A2)
$$v_{w}=L\;\left(\Delta \pi -P\right)$$

*Rate of change in water volume* = *water uptake rate* − *transpiration rate*

(A3)
$$\;P=\frac{L\;\Delta \pi +\phi P_{C}\;}{\phi +L}$$

*Turgor pressure* = *constant*

This set of global biophysical equations are obtained from Equations (1)–(3) by making *P* a constant, thus d*P*/d*t* = 0. And if the relative transpiration rate term, *v*_T_, is neglected or zero, then the same set of equations derived by Lockhart [11] are recovered, i.e., Equations (A1)–(A3).

### Appendix D.2. Set of Dimensionless Global Biophysical Equations

These are Equations (1)–(3) in dimensionless form [5,7,8].

(A4) $$\;v_{\mathrm{cw}}{}^{*}=\Pi_{\mathrm{pv}}\;\left(P^{*}-1\right)+\Pi_{\mathrm{ev}}\;{\frac{\;dP^{*}}{dt^{*}}}_{\;}{}^{\;}$$

(A5) $$\;v_{w}{}^{*}=\Pi_{\mathrm{wv}}\;\left(\Delta \pi^{*}-P^{*}\right)-\Pi_{\mathrm{Tv}}\;\;v_{T}{}^{*}$$

(A6) $$\frac{\;dP^{*}}{dt^{*}}=\Pi_{\mathrm{we}}\;\left(\Delta \pi^{*}-P^{*}\right)-\Pi_{\mathrm{Te}}\;\;v_{T}{}^{*}-\Pi_{\mathrm{pe}}\;\left(P^{*}-1\right)$$

An asterisk (*) designates the dimensionless variables in Equations (A7)–(A9). The dimensionless coefficient (Π parameter) preceding each term represents the dimensionless magnitude of that term. The interpretation of the dimensionless Π parameters is expressed in terms of the biophysical variables and as ratios of biophysical processes [5,6,7,8]. For example, the first subscript, w, of the “Π_wv_” parameter refers to the numerator (relative volumetric water uptake rate) and the second subscript, v, refers to the denominator (relative volumetric steady growth rate, *v*_s_).

$$\Pi_{\mathrm{wv}}=\left(\frac{L\;\;P_{C}}{\;v_{s}}\right)=\left(\frac{\;relative\;volumetric\;water\;uptake\;rate}{\;relative\;volumetric\;growth\;rate}\right)$$

$$\Pi_{\mathrm{Tv}}=\left(\frac{v_{\mathrm{sT}}\;\;}{v_{s}\;}\right)=\left(\frac{\;relative\;volumetric\;transpiration\;rate}{\;relative\;volumetric\;growth\;rate}\right)$$

$$\Pi_{\mathrm{pv}}=\left(\frac{\;\phi \;P_{C}}{v_{s}}\right)=\left(\frac{\;relative\;volumetric\;plastic\;\;deformation\;rate\;of\;the\;wall}{\;relative\;volumetric\;growth\;rate}\right)$$

$$\Pi_{\mathrm{ev}}=\left(\frac{\;P_{C}}{\varepsilon }\right)=\left(\frac{\;relative\;volumetric\;elastic\;\;deformation\;rate\;of\;the\;wall}{\;relative\;volumetric\;growth\;rate}\right)$$

$$\Pi_{\mathrm{we}}=\left(\frac{\varepsilon \;L\;}{v_{s}}\right)=\left(\frac{relative\;volumetric\;\;water\;uptake\;rate}{relative\;volumetric\;elastic\;\;deformation\;rate\;of\;the\;wall}\right)$$

$$\Pi_{\mathrm{Te}}=\left(\frac{\varepsilon \;\;v_{\mathrm{sT}}\;}{P_{C}\;v_{s}}\right)=\left(\frac{\;\;relative\;volumetric\;transpiration\;rate}{relative\;volumetric\;elastic\;\;deformation\;rate\;of\;the\;wall\;}\right)$$

$$\Pi_{\mathrm{pe}}=\left(\frac{\varepsilon \;\phi \;}{v_{s}}\right)=\left(\frac{\;relative\;volumetric\;plastic\;deformation\;rate\;of\;the\;wall}{relative\;volumetric\;elastic\;\;deformation\;rate\;of\;the\;wall}\right)$$

### Appendix D.3. Set of Global Biophysical Equations for Plant Tissue When the Pressure in the Cell Wall Is Not Zero

The cell wall is part of the apoplasm pathway in plant tissue and the pressure within may be different than the atmospheric pressure. For Equations (A7)–(A9), the pressure inside the plasma membrane is defined as, *P*_I_, and the pressure in the cell wall is defined as, *P*_A_ [3]. Both *P*_I_ and *P*_A_ are gauge pressures relative to the atmosphere, which is defined to be zero. *P*_I_ is measured with the pressure probe and *P*_A_ is measured with the pressure chamber. The osmotic pressure difference is, Δ*π* = *π*_I_ − *π*_A_, where *π*_I_ is the osmotic pressure inside the plasma membrane and *π*_A_ is the osmotic pressure within the cell wall. The transpiration term in Equations (A8) and (A9) are zero because the cells are inside the tissue and do not transpire. The hydraulic conductance, *L*, is defined as, *L* = *L*_p_ (*A*/*V*).

(A7) $$v_{\mathrm{cw}}=\phi \;\left[\left(P_{I}-P_{A}\right)-P_{C}\right]+\left(\frac{1}{\varepsilon }\right)\;\left(\frac{\;dP_{I}}{dt}-\frac{\;dP_{A}}{dt}\right)$$

*Rate of change in cell wall chamber volume* = *plastic deformation rate* + *elastic deformation rate*

(A8) $$v_{w}=L\;\left[\Delta \pi -\left(P_{I}-P_{A}\right)\right]$$

*Rate of change in water volume* = *water uptake rate*

(A9) $$\frac{\;dP_{I}}{dt}+\varepsilon \;\left(\phi +L\right)\;P_{I}=\varepsilon \;\left(\;L\;\;\Delta \pi +\phi \;P_{C}\;\right)+\varepsilon \;\left(\phi +L\right)\;P_{A}+\frac{\;dP_{A}}{dt}\;$$

*Equation describing the rate of change in turgor pressure, P_I_, and rate of change in pressure in apoplasm, P_A_.*
